# The Influence of Cognition, Anxiety and Psychiatric Disorders over Treatment Adherence in Uncontrolled Hypertensive Patients

**DOI:** 10.1371/journal.pone.0022925

**Published:** 2011-08-09

**Authors:** Úrsula Jacobs, Mauro S. De Castro, Flávio D. Fuchs, Maria Beatriz C. Ferreira

**Affiliations:** 1 Postgraduate Program in Medical Sciences, School of Medicine, Universidade Federal do Rio Grande do Sul (UFRGS), Porto Alegre, Brazil; 2 Department of Drug Production and Quality Control, School of Pharmacy, Universidade Federal do Rio Grande do Sul (UFRGS), Porto Alegre, Brazil; 3 Division of Cardiology, Hospital de Clínicas de Porto Alegre, Porto Alegre, Brazil; 4 Division of Cardiology, Hospital de Clínicas de Porto Alegre, Universidade Federal do Rio Grande do Sul (UFRGS), Porto Alegre, Brazil; 5 Pharmacology Department, Health Basic Sciences Institute, Universidade Federal do Rio Grande do Sul, Porto Alegre, Brazil; 6 Postgraduate Program in Medical Sciences, Hospital de Clínicas de Porto Alegre, Porto Alegre, Brazil; University of Modena and Reggio Emilia, Italy

## Abstract

**Background:**

Poor adherence is estimated to cause 125 thousand deaths per year and is linked to 10% of all hospital stays in the U.S. Up to one third of elderly hypertensive patients don't have adherence, which is responsible for high proportion of hospitalizations. Hypertension is also related to poor performance in tests that assess cognitive functions. On the other hand, poor cognitive performance is associated with low adherence to treatment.

**Objective:**

To assess the association between cognitive function, anxiety and psychiatric disorders with adherence to drug treatment in patients with hypertension.

**Methodology and Principal Findings:**

This a cohort studies with 56 adult patients with uncontrolled hypertension who participated of all meetings of a pharmaceutical intervention in a randomized clinical trial of pharmaceutical care. Cognitive function was measured by the Mini Mental State Examination (*Mini-mental*). The memory was measured by digit and word spans, tower and church shadow test, short story test and metamemory. Anxiety and psychiatric disorders were evaluated by the State Trace Anxiety Inventory and the Self-Report Questionnaire, respectively. The participants were classified as adherent or non-adherent to the drug treatment, according to the identification of plasma levels of hydrochlorothiazide. All non-adherent patients (n = 12) and 35 out 44 (79.5%) patients with adherence to treatment had at least one memory test with an altered score (*P* = 0.180). Participants with an unsatisfactory score in the Mini-mental had six-fold higher risk of non-adherence to treatment when compared to those with a normal score (RR = 5.8; CI 95%: 1.6–20.8; *P* = 0.007). The scores of anxiety and psychiatric disorders were not associated with adherence to the pharmacological treatment.

**Conclusion:**

Cognitive deficit impairs adherence to drug therapy and should be screened as part of a program of pharmaceutical care to improve adherence to treatment.

## Introduction

Non-adherence to medical regimens is a worldwide problem. According to the World Health Organization (WHO), the average rate of non-adherence in patients with chronic disease is 50% in developed countries [1]. This may be related to poor therapeutic results and determines the wasting of billions of dollars per year in the United States [2]. Poor adherence is estimated to cause 125 thousand deaths per year and is linked to 10% of all hospital stays in the U.S. [2].

Worldwide, high blood pressure is estimated to cause 7.1 million deaths per year [3]. Hypertension, a chronic condition responsible for high proportion of hospitalizations in Brazil [4], is related to poor performance in tests that assess cognitive functions [5], [6]. This pathology deranges memory and attention and can arouse feelings of anxiety and depression [7], due to its diagnosis as well as its treatment. Poor cognitive performance is associated with low adherence to treatment [8], [9], and it has been suggested that education plays an important impact in cognitive function and adherence [9].

Cognition is based on the successive processing of information and the recognition of this succession [10]. Emotions, level of consciousness and mood modulate the perception of different stimuli to which we are exposed daily [11]. Since memory influences several aspects of life, and communication is the key element to well-being and survival [11], their maladjustment can compromise the whole performance of individuals.

The objective of this study is therefore to investigate the hypothesis that cognitive function, anxiety and psychiatric disorders are associated with adherence to drug therapy in patients with uncontrolled hypertension.

## Methods

This is a cohort study nested in a randomized clinical trial, which reported the efficacy of pharmaceutical care to improve blood pressure (BP) control [12]. The study was carried-out in the Outpatient Hypertension Clinic of the Cardiology Division of the Hospital de Clínicas de Porto Alegre (Porto Alegre, RS Brazil). Patients had uncontrolled blood pressure. The factors studied – cognition, memory, anxiety and psychiatric disorders – were analyzed using specific instruments. Performance in the Mini-Mental was the main exposure variable, since it assesses the overall cognitive function. The outcome “adherence to treatment” was determined by the presence of plasma levels of hydrochlorothiazide (qualitative analysis), prescribed for all patients, using high performance liquid chromatography (HPLC Shimadzu®) coupled to the Micromass® mass spectrometer. Blood sampling was collected in the last consultation.

Fifty-six adult patients over 18 years of age, with uncontrolled hypertension (BP ≥140/90 mmHg), were included in the analysis. These subjects were under drug treatment with hydrochlorothiazide alone or in association, and were submitted to a serial of tests of cognition, anxiety and psychiatric disorders. Patients were followed-up for six months and the tests were applied by a pharmacist in one of the five consultations in the follow-up.

### Measurement instruments

Mini Mental State Exam – *Mini-mental* (MM) [13] encompasses issues of temporal and spatial orientation, data registry, attention and calculation, evocation of registered data and language, each one with a specific evaluation. The maximum score is 30 points. The cut-off point for people with 4 years or less of education is 17, and for those with more than 4 years of education, 24 points [14].

Word span [15] assesses the immediate (WSI) and short-term (WSST) memory and consists of the reading of a list of ten words to the patient, who must repeat them immediately (WSI) and after a few minutes (WSST), receiving 1 point for each correct word, regardless of order. The cut-off point for WSI is 4 and WSST is 3.

Digit span [15] evaluates operational memory, and is a verbal test that comprises seven numerical series. The first has 3 digits, and one digit is added to each series until the final one, which has 9 digits. The maximum score is 14 points, and the minimum expected is 5.

Tower and church shadow test (TCS) [16] assesses non-verbal memory. Ten shadows are shown for 5 seconds each to the patient and then he/she must locate each one on a sheet of paper with 44 different shadows, marking them with rings within the period of one minute.

Short story [15] evaluates logic memory by the slow and paced reading of a story with ten items to be remembered. The patient must tell the story twice, once immediately after hearing it and the other after ten minutes.

Memory Self-Assessment Scale – Metamemory [17] - evaluates the perception that individuals have regarding their own memory. It is comprised by ten questions that refer to the memory functions at the moment of assessment, compared to the period before the use of medication or the attendance to the medical service.

State Trace Anxiety Inventory (STAI) [18] evaluates anxiety state and trace. It is divided in two parts. The first part refers to the anxiety-state, when the patient responds according to what he/she is feeling at the moment of the assessment. In the second part the patient must answer according to how he/she generally feels (anxiety-trace).

Self Report Questionnaire (SRQ) [19] evaluates the suspicion of psychiatric disorders through questions about the life of the patient. The Portuguese version of the SRQ 20 determines the cut-off point for not presenting psychotic morbidity at ≤8 for women and ≤6 for men [20].

The instruments used in this study were previously validated for the Portuguese language and were applied by trained pharmacists.

The original study [12] and this study were approved by the Ethics Committee of Hospital de Clínicas of Porto Alegre, which is accredited by the Office of Human Research Protections as an Institutional Review Board, and every participant signed a term of informed consent. The duration of the trial was of six months.

### Statistical analysis

Adherence to pharmaceutical treatment was categorized as adherence or non-adherence according to the presence of hydrochlorothiazide in the plasma. Continuous variables were analyzed using Student's t test, whereas categorical variables were tested by the chi-square or Fischer's exact tests, when appropriate. Poisson regression analysis was employed, adjusting for robust variables, in order to eliminate the effect of possible confounding. Variables that presented a value of *P*<0.20 in the univariate analysis of factors studied (MM e WSST) with baseline characteristics or with outcome were entered in the multivariate model, and the treatment in the original trial (pharmaceutical intervention or placebo intervention) was forced in the model.

Years of education were stratified to analyze the Mini-mental (MM) performance. Mini-mental was categorized as normal or altered, according to the cutoffs proposed by the scale (for people with 4 years or less of education is 17, and for those with more than 4 years of education, 24 points). Using a convenience sample, a power of 83% was obtained to show a difference of proportion in categorical scores in the MM, between the individuals who adhered to treatment and those who did not; with a probability of error α of 5%. Analyses were carried out using the SPSS program, version 16.0 for Windows. Sensitivity and specificity of the MM and WSST instruments were also calculated as indicators of adherence to treatment.

## Results

Seventy-one patients participated in the randomized in the clinical trial. Of these, 56 performed all activities and were included in this study.

Baseline characteristics were similar between participants who adhered and did not adhere to pharmacological treatment (Table 1). Although there was no significant statistical difference, the absolute proportion of patients with long lasting and uncontrolled hypertension and with diabetes was higher among the non-adherent group. More than 40% of patients in both groups presented white coat phenomenon.

**Table 1:** Baseline characteristics of the patients, according to the adherence to treatment, measured by plasmatic levels of hydrochlorothiazide (mean ± SD or n[%], when appropriate).

Characteristics	Adherence (n = 44)	Non-adherence (n = 12)	*P*
Women	29 (65.9)	7 (58.3)	0.737
Age	62.5±9.1	58.7±11.3	0.229
Years of education	6.7±4.4	6.67±3.0	0.991
Pharmacotherapeutic follow-up	21 (47.7)	5 (41.7)	0.963
Duration of hypertension >10 years	33 (75.0)	11 (91.7)	0.427
Individuals with uncontrolled hypertension >2 years	26 (59.1)	9 (75.0)	0.503
Patients with white coat syndrome^a^	20 (45.5)	5 (41.7)	0.999
Diabetes mellitus	16 (36.4)	6 (50.0)	0.508
No. of medications in use	6.4±2.3	6.4±3.1	0.993
No. of other illnesses	3.6±1.7	3.2±1.3	0.474
Office systolic blood pressure at baseline evaluation	165.3±19.2	161.5±12.1	0.518
Office diastolic blood pressure at baseline evaluation	88.5±11.7	88.2±11.1	0.935

a Diagnosed by Ambulatory Blood Pressure Monitoring.

All patients who did not adhere to treatment showed at least one altered test for immediate memory and one altered test for short-term memory. Individual analysis of the other instruments used to assess memory and self-perception of memory did not reveal statistically significant differences, despite that the absolute performance tended to be worst in non-adherent patients.

The proportion of patients with normal score in the MM was greater in the adherence group (81.4% *vs* 33%, *P* = 0.003). This result could also be seen in the WSST (45.5% *vs* 8%, *P* = 0.021). The presence of altered MM and WSST were positively associated with non-adherence by the adjusted residue of the chi-square analysis.

The Poisson regression analysis, adjusting for age, baseline systolic and diastolic blood pressure and pharmacotherapeutic intervention, is shown in Table 2. Individuals with altered Mini-mental had six-fold greater risk of not adhering to treatment (RR = 5.8; CI95%: 1.6–20.8, *P* = 0.007). For word span short-term there was a trend for significance (RR = 6.1; CI95% 0.9–43.3, *P* = 0.071).

**Table 2:** Poison regression analysis: Mini-mental (MM) and word span short-term (WSST) as risk factor exposition of non-adherence.

Risk factor exposition	Relative risk (RR)*	CI 95%	*P*
Altered MM	5.8	1.6–20.8	0.007
Altered WSST	6.1	0.9–43.3	0.071

*Adjusting for age, baseline systolic and diastolic blood pressure and pharmacotherapy intervention.

MM showed sensitivity of 67% and specificity of 81% to predict non-adherence, using 17 as the cut-off point for education ≤4 years and 24 for education >4 years. The chance of having an altered MM was 3.6 times greater among those who did not adhere to the pharmacological treatment. The probability of non-adherence having normal MM was 10%.

WSST showed greater sensitivity for non-adherence than MM (91%), and lower specificity (45%), using 3 as the cut-off point. The probability of having an altered WSST was 1.7 times higher among those who did not adhere to treatment, compared to those who did adhere. The probability of non-adherence with normal WSST was 4.8%.

When it was considered the presence of alteration in both tests, the specificity remained similar to the MM alone, but there was reduction in the sensitivity (25%) and in the likelihood ratio (1.0). Considering the presence of at least one altered test, there was an increase in the sensitivity (83%) and reduction in the specificity (38%) and likelihood ratio (1.4).

The proportion of patients with evidence of anxiety and psychiatric disorder in any of the several tests employed was not statistically different between the adherence and non-adherence groups (data not shown).

## Discussion

In this study of patients with hypertension submitted to a clinical trial of pharmaceutical care it was possible to identify an independent association between cognitive deficit and low adherence. Anxiety and psychiatric disorders were not associated with low adherence.

The Mini-mental was the only test that showed an independent association between deficit of cognition and low adherence to treatment. This test showed also some power to detect (sensitivity of 66%) and rule out (specificity of 81%) lack of adherence in this context. A study conducted in Japan found a sensitivity of 75% and a specificity of 81.5% for non-adherence with MM, using 26 as the cut-off point, for patients hospitalized with diabetes and intolerance to glucose [21].

While some studies use MM as screening process for the selection of patients for verification of the loss of cognitive functions [22], [23], we recommend the use of this instrument as a determiner of the approach of the pharmacist with regard to the patient.

Furthermore, non-adherent patients had a worse performance in other tests, despite the absence of statistical significance. A beta error can not be discarded. Interestingly, the issue of memory was not similar between the tests. This may be due to the different domains treated in each test – auditive *versus* visual memory, memory of isolated symbols (as words and numbers) *versus* memory of more complex context (as a short story), as well as the role of each one of these domains in the posterior evocation of the medical recommendations.

The digit span, WWST and short story, although evaluating immediate and short-term memory, showed no difference between the groups being compared, possibly because the scores obtained were so poor (equal to or less than the minimum expected) for all patients, regardless of the group.

In the case of the short story, patients need to recall ten points from an entire situation, not only words, and the average scores for immediate and short-term memory were 50% and 37%, respectively. This test could represent a medical recommendation situation where the patient is told a series of things he must follow. Using this scenario, it is necessary to consider the sufficiency of immediately, remembering only half of what was said and about the fitness of the 1/3 law [24] regarding the memory of information minutes after hearing it. We may have revealed the need to use more than verbal communication tool to instruct patients, perhaps combining this with support material whenever necessary.

The tower and church shadow test, which evaluates immediate and short-term visual memory, did not reveal any association with adherence to treatment. Patient performance was good, recalling 70% of all shadows right after visualization and 60% 15 minutes later, indicating good visual memory in this sample. Therefore, the interventions that use this memory could probably be used to instruct these patients.

The Memory Self-Assessment Scale was hard to apply and probably quite complex for understanding. Therefore, this instrument is not appropriate for assessing non-adherence.

The findings for the tests that evaluated anxiety and psychiatric disorders did not contribute to identify reasons for non-adherence to drug treatment. A beta error cannot be fully discarded. These data differ from Campos' findings, in a report that describes symptoms of anxiety as an independent risk factor for non-adherence [25] in patients with AIDS and the Kuhl' findings, in a study that describes anxiety was related to lower adherence to several important risk-reducing recommendations after myocardial infarction [26].

Pharmaceutical care has been used to improve adherence to treatment of hypertension [12], [27]. The evaluation of cognition by the Mini-mental test and eventually the word span short-term may help to identify patients at higher risk of low adherence to treatment in the pharmacist consultation.

Our study has some limitations that deserve mention. In view of the criteria of selection, which included the necessity to understand the request for consent, diminished the probability of having enrolled participants with relevant psychiatric disorders or high anxiety. Despite the small sample size, however, the association of cognitive deficit and lost of memory emerged, but the absence of some associations may be ascribed to a beta error. On the other side, the diagnosis of adherence by plasma levels of hydrochlorothiazide and the full evaluation of cognitive performance are strengths of our investigation.

In conclusion, cognitive impairment reduces the adherence to treatment of patients with hypertension. The screening for such abnormalities should be considered in the pharmaceutical care of patients, in order to minimize its influence upon the adherence to the treatment.
